# HCMV-encoded miRNAs in an EV-enriched plasma particle fraction as candidate biomarkers of HCMV DNAemia

**DOI:** 10.3389/fmicb.2026.1913023

**Published:** 2026-09-24

**Authors:** Wanqing Zhou, Dongmei Niu, Hui Xie, Shuo Gao, Chunni Zhang, Han Shen, Cheng Wang

**Affiliations:** 1 Department of Laboratory Medicine, Nanjing Drum Tower Hospital, Affiliated Hospital of Medical School, Nanjing University, Nanjing, China; 2 Department of Laboratory Medicine, Nanjing Hospital of Chinese Medicine Affiliated to Nanjing University of Chinese Medicine, Nanjing, China; 3 State Key Laboratory of Analytical Chemistry for Life Science, Department of Clinical Laboratory, Nanjing University School of Medicine, Jinling Hospital, Nanjing University, Nanjing, China

**Keywords:** biomarker, EV-enriched plasma fraction, extracellular vesicle, HCMV DNAemia, human cytomegalovirus, microRNA, viral miRNA

## Abstract

**Background:**

Extracellular vesicle-enriched plasma particle fractions may contain viral and host RNA species that reflect infection-associated changes. However, whether HCMV-encoded miRNAs detected in circulating particle fractions are associated with HCMV DNAemia remains incompletely understood. This study analyzed selected HCMV-encoded miRNAs in an ExoQuick-derived EV-enriched plasma precipitate from patients with HCMV DNAemia and DNA-low, IgG-positive controls.

**Methods:**

Plasma samples were collected from 41 patients with HCMV DNAemia, defined as HCMV DNA > 500 IU/mL, and 43 DNA-low, IgG-positive controls, defined as HCMV DNA < 500 IU/mL with HCMV IgG positivity and IgM negativity. Eight HCMV miRNAs were quantified using stem-loop RT-qPCR. HCMV DNA was measured by qPCR. A longitudinal series of 30 samples from seven treated patients was analyzed exploratorily.

**Results:**

TEM and NTA showed that the ExoQuick-derived preparations contained vesicle-like nanoparticles with round or cup-shaped morphology and particle-size distributions compatible with small EV-enriched preparations. Among the eight candidate HCMV miRNAs, hcmv-miR-US4-5p and hcmv-miR-US25-2-5p remained significantly increased in the HCMV DNAemia group after false-discovery-rate correction. Hcmv-miR-US25-1-3p and hcmv-miR-UL36 showed nominal increases but did not remain significant after correction. Exploratory ROC analyses showed modest AUC values of approximately 0.64–0.73, with sensitivities below 45%. Longitudinal patterns were heterogeneous among the seven treated patients.

**Conclusion:**

Selected HCMV-encoded miRNAs, particularly hcmv-miR-US4-5p and hcmv-miR-US25-2-5p were increased in an ExoQuick-derived EV-enriched plasma particle fraction from patients with HCMV DNAemia. These findings suggest potential exploratory auxiliary biomarker value, but definitive assignment to EV-encapsulated cargo and their utility for disease monitoring require further validation using EV-marker analysis, nuclease/protease protection assays, broader miRNA profiling, and independent validation cohorts are required.

## Background

Extracellular vesicles (EVs) are nanoscale, membrane-bound vesicles secreted by virtually all cell types and are found in most bodily fluids (Safeer and Murali, 2025). They carry diverse cargoes, including proteins, nucleic acids, lipids, and metabolites. The molecular cargo of EVs often reflects the physiological or pathological state of their parent cell. By transferring their biomolecular cargo to recipient cells, EVs facilitate intercellular communication (O'Brien et al., 2020).

EV biogenesis is connected to endosomal and secretory pathways, and these pathways can be influenced by viral infection in experimental systems (Post et al., 2025). Herpesviruses have been reported to interact with cellular membrane-trafficking machinery during infection. These observations provide a general biological rationale for studying viral RNA species in circulating particle fractions.

Viruses, as obligate intracellular pathogens, exploit host cellular machinery for survival and propagation. Following infection, viral proteins and nucleic acids have been reported in host-derived vesicles or extracellular particle fractions in several experimental systems. Such virus-associated extracellular particles may participate in host-pathogen interactions, although the precise carrier subtype and functional relevance can vary by virus, cell type, and isolation method (Streck et al., 2020). Consequently, detecting virus-specific molecules within EVs holds promise for developing diagnostic markers and therapeutic targets (Safeer and Murali, 2025; Meckes, 2015). MicroRNAs (miRNAs), a class of small non-coding RNAs, can be associated with EVs or other extracellular RNA carriers and may participate in intercellular communication (Liu et al., 2019; Groot and Lee, 2020). This phenomenon has been described in experimental models of Epstein–Barr virus infection, where EBV-encoded miRNAs associated with EVs were reported to suppress target gene expression in recipient cells (Gallo et al., 2017). Subsequent studies on Kaposi’s sarcoma-associated herpesvirus (KSHV) and human cytomegalovirus (HCMV) have similarly demonstrated that viral miRNAs packaged into EVs exert specific biological functions, highlighting a critical mechanism of viral pathogenesis (Yogev et al., 2017; Mohammad et al., 2017).

Human cytomegalovirus (HCMV), a member of the *Herpesviridae* family, is a large double-stranded DNA virus transmitted through various routes, including sexual contact, breastfeeding, blood transfusion, and organ transplantation. Accumulating evidence indicates that HCMV encodes numerous miRNAs that modulate both host and viral gene expression to regulate immune escape, cell cycle progression, and the maintenance of latency (Zhang L. et al., 2020). *In vitro* studies have suggested that HCMV-encoded miRNAs may be transferred to uninfected cells via extracellular vesicle-related pathways. Clinical studies have also reported associations between HCMV miRNAs detected in serum EV-enriched fractions, including hcmv-miR-US25-1-5p and hcmv-miR-UL112-3p, and pediatric liver injury. However, the precise extracellular carriers and intravesicular localization of these miRNAs require further validation (Zhang J. et al., 2020). Although latently infected cells do not release infectious virions, experimental studies suggest that HCMV-encoded miRNAs can be detected in extracellular vesicle-related fractions during latency, potentially contributing to immune modulation. The carrier identity and functional effects of these miRNAs in clinical samples remain to be fully defined (Ding et al., 2017). Therefore, investigating the transport and function of HCMV-encoded miRNAs is essential for elucidating their roles in disease pathogenesis.

Our previous work revealed distinct expression profiles of HCMV-encoded miRNAs in the peripheral blood of patients with HCMV viremia compared to those with latent infection (Zhou et al., 2020). However, how these miRNAs are transported in circulation during HCMV viremia remains unclear. The present study was designed as a targeted descriptive extension of that work. We quantified eight preselected HCMV miRNAs in an ExoQuick-derived plasma particle precipitate from patients with HCMV DNAemia and DNA-low, IgG-positive controls and explored whether these miRNA signals were associated with HCMV DNAemia.

## Materials and methods

### Participants and study design

A total of 84 participants with evidence of prior HCMV infection were enrolled. Participants were assigned to the HCMV DNAemia group (*n* = 41) when HCMV DNA was >500 IU/mL, with HCMV IgG positivity and HCMV IgM negativity. DNA-low, IgG-positive controls (*n* = 43) were defined as participants with HCMV DNA < 500 IU/mL, HCMV IgG positivity, and HCMV IgM negativity. The 500 IU/mL threshold corresponded to the lower quantitative threshold/clinical reporting cut-off of the HCMV DNA assay used in our laboratory. Samples below this threshold were reported as <500 IU/mL and were not assigned a numerical viral-load value for group comparison. An additional longitudinal cohort consisted of 30 serial samples collected from seven patients undergoing antiviral therapy with ganciclovir. Their underlying diseases included acute lymphoblastic leukemia (*n* = 3), acute myeloid leukemia (*n* = 1), autoimmune hemolytic anemia (*n* = 1), diffuse large B-cell lymphoma (*n* = 1), and systemic lupus erythematosus (*n* = 1). All clinical data and blood samples were obtained after participants provided written informed consent, in accordance with protocols approved by the Ethics Committee of Nanjing Drum Tower Hospital. Demographic and clinical data, including age, sex, diagnosis, and HCMV, HCV, and HIV status, were recorded (Table 1).

**Table 1:** Demographic and clinical features of patients in the present study.

Variable	DNAemia group (*n* = 41)	Control (*n* = 43)	*p* values
Age, years^†^	53.0 ± 19.3	51.2 ± 18.0	0.62^‡^
Sex, *n*			0.52^§^
Male	21 (51.2%)	25 (58.1%)	
Female	20 (48.8%)	18 (41.9%)	
HCMV DNA Level^¶^	1,610 (804.5, 10,350)	–	
Anti-HCMV IgG (IU/mL) ^†^	1.83 ± 0.83	1.74 ± 0.92	0.41 ^‡^
Anti-HCMV IgM, *n*			
Positive	0 (0%)	0 (0%)	
Negative	41 (100%)	43 (100%)	
HCV, *n*			
Positive	0 (0%)	0 (0%)	
Negative	41 (100%)	43 (100%)	
HIV, *n*			
Positive	0 (0%)	0 (0%)	
Negative	41 (100%)	43 (100%)	
*Diagnostic*			
Leukemia, *n*			0. 80^§^
Yes	11 (26.8%)	10 (23.3%)	
No	30 (73.2%)	33 (76.7%)	
Autoimmune diseases, *n*			0.40 ^§^
Yes	9 (22.0%)	6 (14.0%)	
No	32 (78.0%)	37 (86.0%)	
Respiratory diseases, *n*			0. 79^§^
Yes	9 (22.0%)	8 (18.6%)	
No	32 (78.0%)	35 (81.4%)	

†, Age data and anti-HCMV IgG are presented as the mean ± SD; ‡, Student *t* test; §, Two-sided χ^2^ test; ¶, HCMV DNA level are presented as median (25% Percentile, 75% Percentile).

Peripheral blood was collected into EDTA anticoagulant tubes and processed within 1 h of collection. Plasma was separated by centrifugation and aliquoted to avoid repeated freeze–thaw cycles. Samples with visible hemolysis were excluded. Plasma aliquots were stored at −80 °C until EV-enriched particle isolation. For each sample, 500 μL of plasma was used for ExoQuick-based precipitation according to the manufacturer’s protocol.

### HCMV DNA load

HCMV DNA levels in peripheral blood leukocytes were quantified using a commercial qPCR kit (DAAN GENE, Guangzhou, China) on an ABI 7500 PCR System (Applied Biosystems, United States), following a previously described protocol (Zhou et al., 2020). Absolute HCMV DNA levels were calculated using a calibrator and are expressed as IU/mL of blood (Ljungman et al., 2019).

### Anti-HCMV IgG and IgM antibody determination

Serum anti-HCMV IgG and IgM antibodies were measured using a commercial ELISA kit (MEDSON, NJ, United States) according to the manufacturer’s instructions and our previous protocol (Zhou et al., 2020). For IgG detection, concentrations were quantified using a calibration curve standardized against the 1st WHO international standard. For IgM detection, samples with an optical density (OD) at 450 nm greater than the cut-off value of 0.25 were considered positive.

### Isolation of an EV-enriched plasma particle fraction

An EV-enriched plasma particle fraction was obtained using the ExoQuick Plasma Prep and Exosome Precipitation Kit (System Biosciences, CA, United States) following the manufacturer’s instructions. ExoQuick is a polymer-based precipitation reagent described by the manufacturer as a simple method for enriching exosome-associated proteins and microRNAs from serum and plasma. Our previous comparative study of commercial exosome isolation kits showed that ExoQuick recovered particles within the expected exosome-size range and enriched EV-associated markers such as CD63 and TSG101, with good miRNA recovery and reproducibility (Ding et al., 2018). Briefly, 500 μL of plasma was treated with 4 μL of thrombin and incubated at room temperature for 5 min, followed by centrifugation at 10,000 × g for 5 min. The supernatant (100 μL) was mixed with 25.2 μL of ExoQuick solution and incubated at 4 °C for 30 min. The mixture was then centrifuged at 1,500 × g for 30 min at 4 °C. After discarding the supernatant, the pellet was subjected to a second centrifugation at 1,500 × g for 5 min to remove residual fluid, and the resulting precipitated particle pellet was retained for downstream analysis. Because precipitation-based methods can co-isolate non-vesicular components, including protein aggregates, lipoproteins, and extracellular ribonucleoprotein complexes, the resulting material is referred to here as an EV-enriched plasma particle fraction. The present study did not include EV-marker analysis or nuclease/protease protection assays to confirm EV identity or intravesicular localization of the detected miRNAs.

### Transmission electron microscopy and nanoparticle tracking analysis

For transmission electron microscopy (TEM), EV-enriched particle pellets were resuspended in 200 μL of phosphate-buffered saline (PBS), adsorbed onto copper grids, and incubated for 5 min. Excess liquid was removed, and the grids were negatively stained with 3% phosphotungstic acid (pH 6.8) for 5 min. After rinsing with double-distilled water and air-drying, micrographs were acquired using a JEM-1011 microscope (JEOL Ltd., Tokyo, Japan). The size distribution and concentration of particles were determined by nanoparticle tracking analysis using an NS300 system (Malvern Panalytical, Worcestershire, United Kingdom). Samples were diluted 1:1000 in PBS, and videos were recorded with camera level, minimum expected particle size, minimum track length, and blur settings set to automatic.

### RNA extraction from EV-enriched plasma particle fractions and RT-qPCR assay

The eight HCMV miRNAs were selected for analysis based on our previous work, which identified them as being abundantly expressed or differentially regulated in the peripheral blood of patients with active versus latent HCMV infection (Zhou et al., 2020). Total RNA was extracted from EV-enriched particle pellets using TRIzol reagent according to a previously described method (Ding et al., 2018). The expression of HCMV-encoded miRNAs was quantified by TaqMan probe-based stem-loop RT-qPCR on a LightCycler^®^ 96 system (Roche Diagnostics, Mannheim, Germany) (Zhou et al., 2020). Briefly, 2 μL of total RNA was reverse-transcribed into cDNA using AMV reverse transcriptase (TaKaRa, Dalian, China) and specific stem-loop RT primers (Applied Biosystems, Foster City, CA, United States). Real-time PCR was performed using hydrolysis miRNA probes. All reactions, including no-template controls, were performed in triplicate. Relative expression was calculated using the 2^−ΔCq^ method, with hsa-miR-16 (MIR16) as the endogenous control.

### Statistical analysis

Statistical analyses were performed using GraphPad Prism 9.0 or SPSS 16.0 software. Continuous variables were summarized as mean ± SD or median with interquartile range, as appropriate. Because miRNA expression values were not normally distributed, between-group comparisons were performed using the Mann–Whitney U test. To account for testing of eight candidate HCMV miRNAs, Benjamini–Hochberg false discovery rate correction was applied. Adjusted *q* values <0.05 were considered statistically significant. Unadjusted *p* values <0.05 with *q* values ≥0.05 were interpreted as nominal differences. Effect sizes for miRNA comparisons were reported as Hodges–Lehmann median differences between groups with 95% confidence intervals. The Chi-square test was used for categorical variables. A *p*-value <0.05 was considered statistically significant. ROC analyses were considered exploratory because the same cohort was used for candidate assessment and performance estimation, and no independent validation cohort was available.

## Results

### Characterization of plasma EV-enriched particle preparations

ExoQuick-derived plasma particle preparations from patients with HCMV DNAemia and DNA-low, IgG-positive controls were characterized by TEM and NTA. TEM revealed vesicle-like particles with round or cup-shaped morphology in both groups (Figures 1A,B). Representative NTA analysis showed that EV-enriched particles from the HCMV DNAemia sample had a major peak at approximately 102.8 nm, with a particle concentration of 8.0 × 10^7^ particles/mL (Figure 1C). The latent infection/control sample showed a major peak at approximately 139.2 nm, with a particle concentration of 2.6 × 10^8^ particles/mL (Figure 1D). These findings indicate recovery of vesicle-like nanoparticles within a size range compatible with small EV-enriched preparations. However, because EV-positive markers, negative contamination markers, and RNase/protease protection assays were not performed, these preparations cannot be considered purified EVs, and miRNA encapsulation cannot be concluded. Because these NTA profiles are representative, future studies with larger sample numbers should perform systematic group-level comparisons of EV size distribution and concentration.

**Figure 1:**
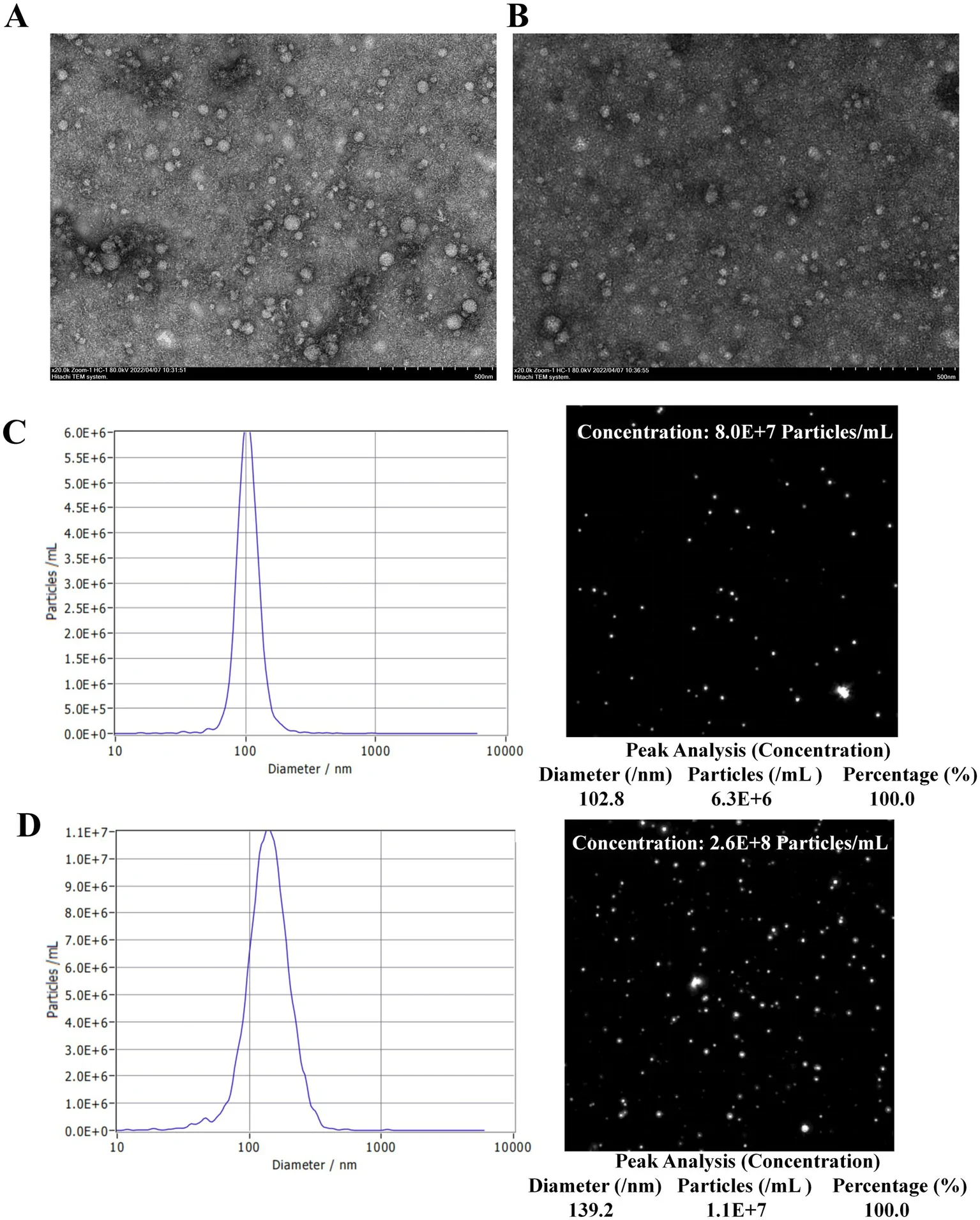
Morphology and size distribution of ExoQuick-derived EV-enriched plasma particle preparations from patients with HCMV DNAemia and DNA-low, IgG-positive controls. **(A)** TEM image of EV-enriched particles from a patient with HCMV DNAemia. **(B)** TEM image of EV-enriched particles from a DNA-low, IgG-positive control subject. **(C)** Representative NTA profile of EV-enriched particles from the HCMV DNAemia sample, showing a major peak at 102.8 nm and a particle concentration of 8.0 × 10^7^ particles/mL. **(D)** Representative NTA profile of EV-enriched particles from the DNA-low, IgG-positive control sample, showing a major peak at 139.2 nm and a particle concentration of 2.6 × 10^8^ particles/mL.

### HCMV miRNA levels in EV-enriched plasma particle fractions

The expression levels of eight preselected HCMV miRNAs were quantified in ExoQuick-derived plasma particle fractions from both patient groups. By unadjusted Mann–Whitney U testing, hcmv-miR-US4-5p, hcmv-miR-US25-2-5p, hcmv-miR-US25-1-3p, and hcmv-miR-UL36 were higher in the HCMV DNAemia group than in DNA-low, IgG-positive controls. After Benjamini–Hochberg correction for testing of eight candidate miRNAs, only hcmv-miR-US4-5p and hcmv-miR-US25-2-5p remained statistically significant. Hcmv-miR-US25-1-3p and hcmv-miR-UL36 were therefore interpreted as nominal findings requiring validation. The remaining four miRNAs showed no significant between-group differences (Table 2; Figure 2). MIR16 showed no significant difference in Cq values between groups, supporting its use for normalization (Figure 2I).

**Table 2:** Expression levels of eight HCMV miRNAs in EV-enriched plasma particle fractions.

miRNA	DNAemia group median, IQR	Control group median, IQR	Hodges–Lehmann median difference, 95% CI	Unadjusted *P*	FDR *q* value
hcmv-miR-US4-5p	0.0103, 0.0188	0.0038, 0.0075	0.0053 (0.0023, 0.0105)	0.0005	0.0040
hcmv-miR-US25-2-5p	0.0825, 0.2442	0.0456, 0.0588	0.0387 (0.0133, 0.0801)	0.0009	0.0036
hcmv-miR-US25-1-3p	0.0806, 0.3537	0.0550, 0.0662	0.0284 (0.0059, 0.0634)	0.0205	0.0547
hcmv-miR-UL36	326.7200, 118.5250	245.0550, 246.4275	96.2100 (4.1600, 250.5300)	0.0418	0.0836
hcmv-miR-UL148D	5.2722, 19.2968	4.2651, 6.8113	1.5739 (−0.1159, 4.0142)	0.0700	0.1120
hcmv-miR-US29-3p	0.0114, 0.0243	0.0065, 0.0140	0.0025 (−0.0003, 0.0065)	0.0908	0.1211
hcmv-miR-US25-2-3p	0.1215, 0.2634	0.0511, 0.1026	0.0295 (−0.0033, 0.0901)	0.0934	0.1067
hcmv-miR-US25-1	1.0605, 3.3590	1.1102, 0.9130	0.0717 (−0.2832, 0.5666)	0.6289	0.6289

Data are presented as median and interquartile range. Effect size represents the Hodges–Lehmann median difference between the DNAemia group and control group with 95% CI. *P* values were calculated using the Mann–Whitney U test. FDR *q* values were calculated using the Benjamini–Hochberg method.

**Figure 2:**
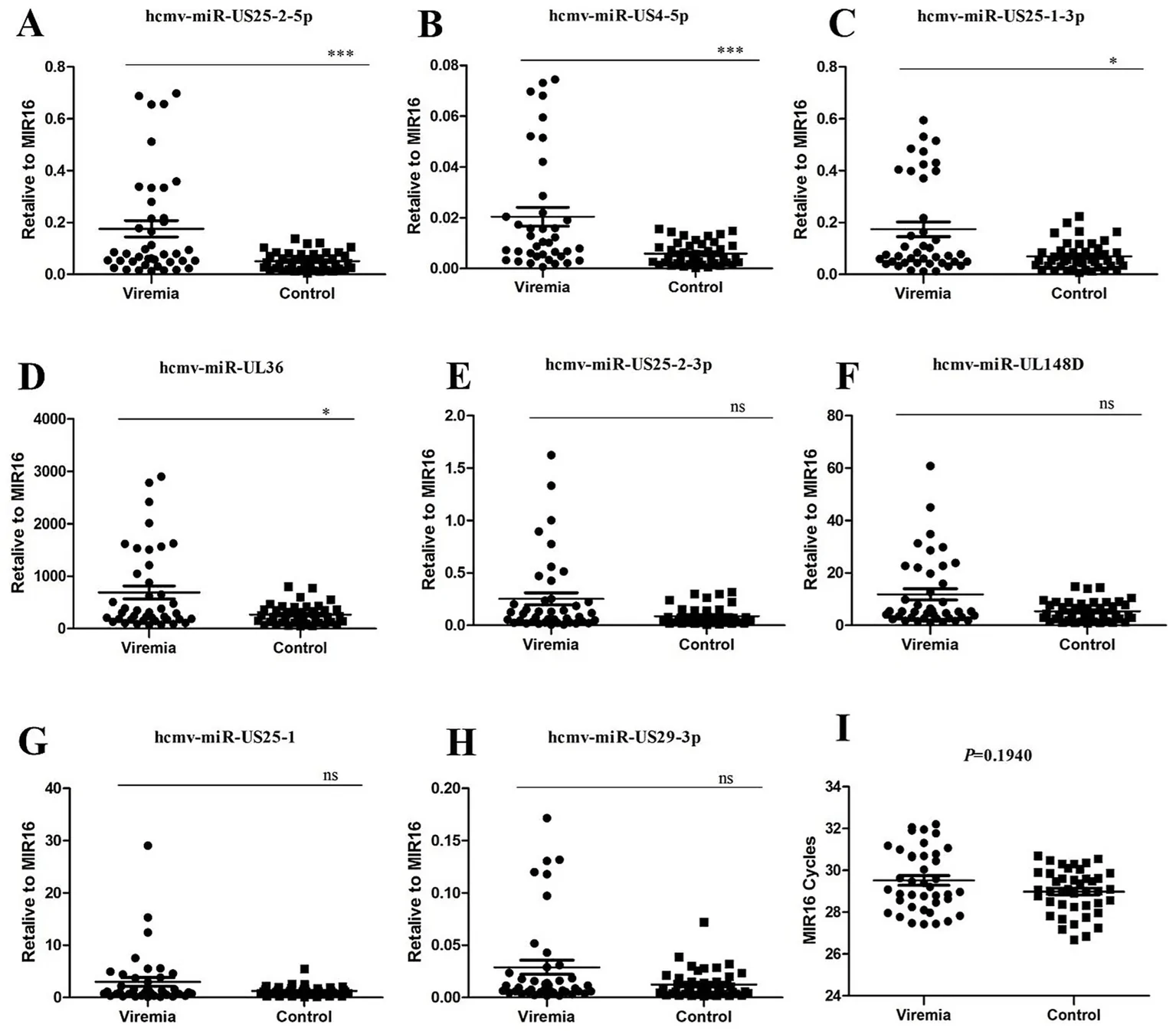
Expression levels of eight HCMV miRNAs in EV-enriched plasma particle fractions detected by RT-qPCR. **(A)** HCMV-miR-US25-2-5p; **(B)** HCMV-miR-US4-5p; **(C)** HCMV-miR-US25-1-3p; **(D)** HCMV-miR-UL36; **(E)** HCMV-miR-US25-2-3p; **(F)** HCMV-miR-UL148D; **(G)** HCMV-miR-US25-1; **(H)** HCMV-miR-US29-3p; **(I)**, *Cq* values of MIR16. Relative expression was calculated using the 2^−ΔCq^ method, normalized to MIR16. Each point represents the mean of triplicate measurements. * *p* < 0.05; *** *p* < 0.001 compared to the control group (Mann–Whitney U test).

### Receiver operating characteristic curve analysis

Exploratory ROC analyses were performed to describe the ability of the measured miRNAs to distinguish the HCMV DNAemia group from DNA-low, IgG-positive controls within the same cohort. The analysis yielded AUCs ranging from 0.64 to 0.73 (Figure 3). The individual performances were as follows: hcmv-miR-US4-5p (AUC: 0.731; 95% CI: 0.620–0.843; sensitivity: 42.1%; specificity: 97.5%), hcmv-miR-US25-2-5p (AUC: 0.722; 95% CI: 0.609–0.835; sensitivity: 39.5%; specificity: 100.0%), hcmv-miR-US25-1-3p (AUC: 0.656; 95% CI: 0.533–0.778; sensitivity: 31.6%; specificity: 97.5%), and hcmv-miR-UL36 (AUC: 0.638; 95% CI: 0.513–0.763; sensitivity: 42.1%; specificity: 90.0%). The individual ROC estimates showed modest diagnostic accuracy with low sensitivities, suggesting limited performance as standalone diagnostic markers and possible future value only as part of a multi-marker panel. Nevertheless, because the same cohort was used for marker selection and ROC estimation, and because no independent validation cohort was available, these analyses should be interpreted as preliminary.

**Figure 3:**
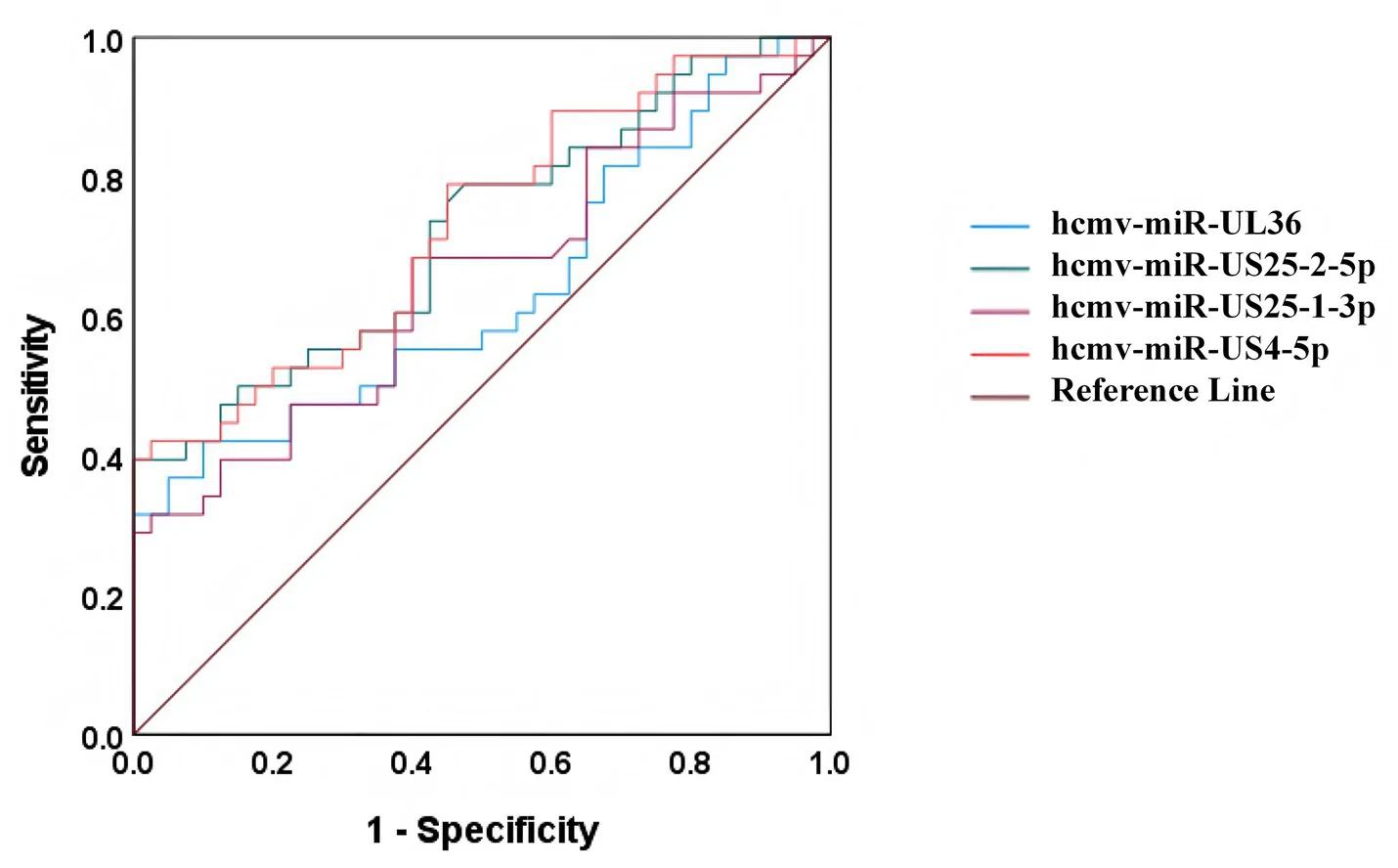
Exploratory ROC analysis of HCMV-encoded miRNAs detected in EV-enriched plasma particle fractions. The analysis shows the ability of each miRNA to distinguish patients with HCMV DNAemia from DNA-low, IgG-positive controls.

### Dynamic changes of HCMV miRNAs in EV-enriched plasma particle fractions during antiviral therapy

To explore temporal changes in particle-fraction-associated HCMV miRNAs during antiviral therapy, we tracked hcmv-miR-US25-1-3p and HCMV DNA in seven patients undergoing antiviral treatment. The results revealed a complex relationship between the two markers (Figure 4). In four patients (Patients 2, 3, 5, and 7), hcmv-miR-US25-1-3p levels in the EV-enriched plasma particle fraction showed an inverse temporal pattern relative to HCMV DNA load during treatment. For instance, as HCMV DNA levels decreased, miRNA levels tended to increase. In contrast, Patient 6 showed a direct correlation, where both markers decreased concurrently. Longitudinal monitoring for Patients 1 and 4 was limited by the study duration. Given the small number of patients, heterogeneous underlying diseases, non-standardized sampling intervals, and inconsistent temporal patterns, these longitudinal observations should be considered hypothesis-generating only.

**Figure 4:**
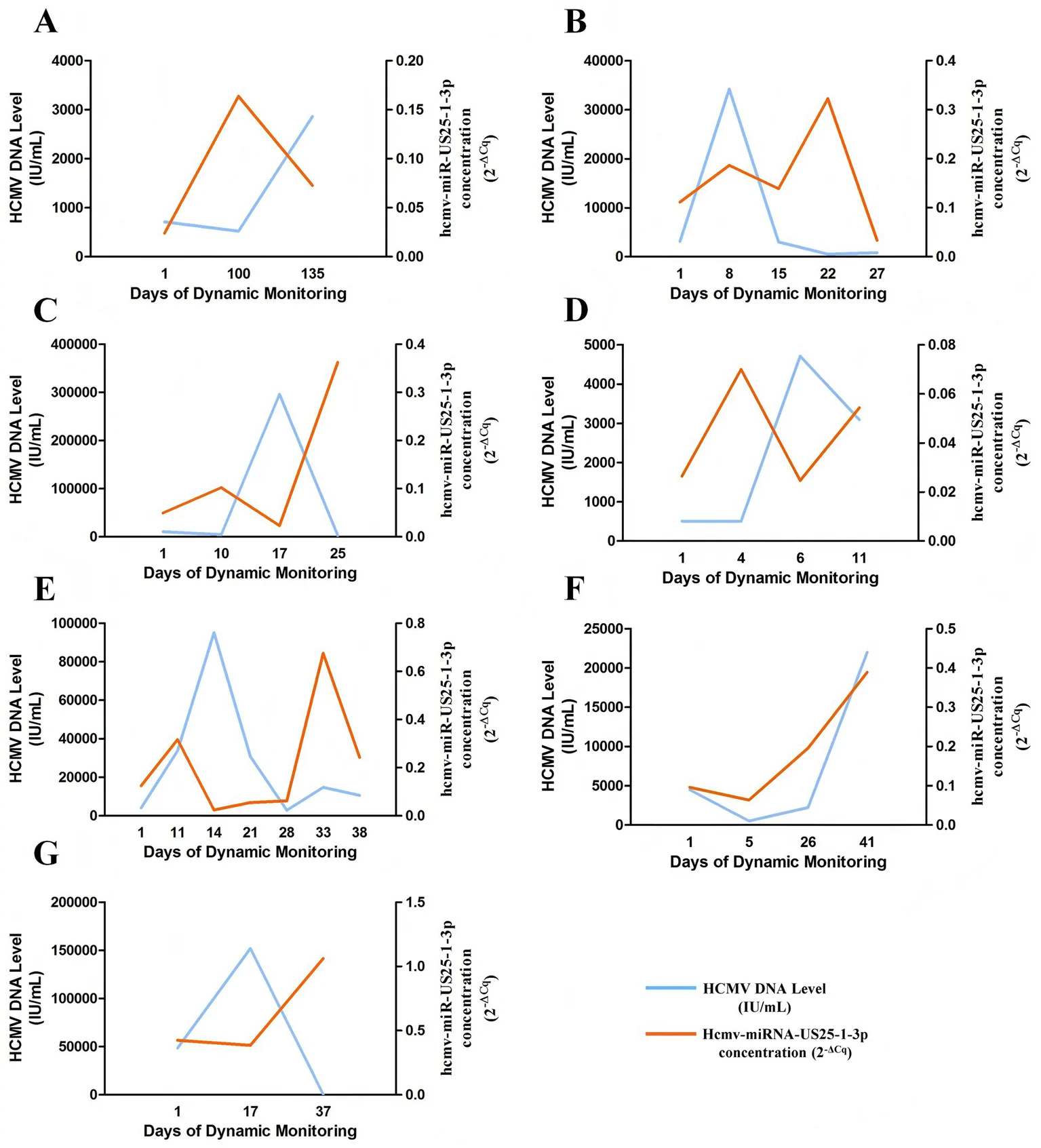
Dynamic changes of HCMV DNA and hcmv-miR-US25-1-3p levels in EV-enriched plasma particle fractions during antiviral treatment. **(A)** Patient 1, diffuse large B-cell lymphoma; **(B)** Patient 2, acute myeloid leukemia; **(C)** Patient 3, autoimmune hemolytic anemia; **(D)** Patient 4, acute lymphoblastic leukemia; **(E)** Patient 5, acute lymphoblastic leukemia; **(F)** Patient 6, acute lymphoblastic leukemia; **(G)** Patient 7, systemic lupus erythematosus. The x-axis represents the time points of dynamic monitoring, with Day 1 as the first day of sample collection. The y-axis represents the levels of HCMV DNA (blue line) and hcmv-miR-US25-1-3p (red line).

## Discussion

HCMV-encoded miRNAs are key regulators of the viral life cycle and host-pathogen interactions, influencing viral latency, replication, and immune evasion (Zhang L. et al., 2020). Our previous work identified specific HCMV miRNAs in peripheral blood that could differentiate between latent and active infection (Zhou et al., 2020). The present study extends those findings by analyzing selected HCMV miRNAs in an ExoQuick-derived EV-enriched plasma particle fraction. We found that hcmv-miR-US4-5p and hcmv-miR-US25-2-5p were increased in the HCMV DNAemia group after correction for multiple testing. Two additional miRNAs, hcmv-miR-US25-1-3p and hcmv-miR-UL36, showed nominal increases but did not remain significant after FDR correction. These findings support their potential as exploratory auxiliary biomarkers, although definitive EV localization and diagnostic utility require further validation.

The present study should also be viewed in relation to our previous circulating-miRNA work. That earlier study identified HCMV-encoded miRNAs in unfractionated circulation that differed between latent and active HCMV infection states (Zhou et al., 2020). The current study does not replace or validate those findings as EV-specific biomarkers. Rather, it provides a targeted follow-up showing that selected HCMV miRNAs can be detected in a precipitation-derived plasma particle fraction and that two of the eight tested miRNAs are associated with HCMV DNAemia after multiple-testing correction.

The detection of HCMV miRNAs in EV-enriched plasma particle fractions may be considered in the broader context of extracellular RNA release during viral infection. EV biogenesis is linked to endosomal and secretory pathways, and experimental studies indicate that herpesviruses can interact with host membrane-trafficking machinery (Post et al., 2025). However, the present study did not directly assess EV biogenesis, endolysosomal function, or vesicle-trafficking pathways. Therefore, our data should not be interpreted as evidence of altered endolysosomal trafficking. Instead, they provide a rationale for future mechanistic studies to determine whether HCMV infection influences extracellular miRNA release through EV-dependent or EV-independent pathways.

The purity of isolated EVs is critical for analyzing their cargo and clinical value, as EVs can share physical properties with viral particles (Li et al., 2022). Although we used a commercial precipitation-based kit that is widely applied in circulating RNA biomarker studies (Chugh et al., 2013; Zhang J. et al., 2020; Bokun et al., 2025), this approach enriches vesicle-like particles but does not yield purified EVs. The ExoQuick product information describes the reagent as a one-step precipitation method for enriching exosome-associated miRNAs and proteins from serum or plasma and highlights its use in biomarker discovery. Moreover, our previous comparative study of commercial exosome isolation kits showed that ExoQuick recovered particles in the expected exosome-size range and enriched EV-associated markers such as CD63 and TSG101, with good miRNA recovery and reproducibility (Ding et al., 2018). Although ExoQuick is suitable for exploratory circulating extracellular RNA studies using limited clinical plasma volumes, the current ExoQuick-derived pellets should not be interpreted as purified EVs without additional validation. Precipitation methods may co-isolate non-vesicular RNA carriers, lipoproteins, protein aggregates, and potentially virus-related particles. Therefore, our data should be interpreted as reflecting HCMV miRNA signals in an EV-enriched plasma precipitate rather than purified host EVs. More stringent approaches, such as size-exclusion chromatography, density-gradient separation, immunoaffinity capture, or nano-flow cytometry, will be required to define the carrier subtype. The increased levels of several miRNAs in the EV-enriched plasma fraction are consistent with our previous findings in whole serum (Zhou et al., 2020), suggesting that these miRNAs are detectable in circulating extracellular particle fractions. However, whether EVs represent the dominant transport mode for these viral miRNAs remains to be determined.

Previous *in vitro* studies have suggested that HCMV-encoded miRNAs can be associated with extracellular vesicle-related pathways (Mohammad et al., 2017; Zhang J. et al., 2020). Our clinical data show that selected HCMV miRNAs are differentially detected in an EV-enriched plasma particle fraction from patients with HCMV DNAemia compared with DNA-low, IgG-positive controls. However, because the present study did not confirm EV encapsulation or carrier identity, these findings should be considered clinical associations rather than *in vivo* confirmation of EV-mediated delivery. For example, Mohammad et al. found that hcmv-miR-US25-1-5p could be delivered to recipient cells via virions in culture (Mohammad et al., 2017), while Zhang et al. demonstrated that both hcmv-miR-US25-1-5p and hcmv-miR-UL112-3p were packaged into EVs from infected cells and were associated with liver injury in infected children (Zhang J. et al., 2020). More recently, specific HCMV miRNAs have been found enriched in placental EVs, implicating them in neurodevelopmental pathways (Martin et al., 2026). Together with prior studies reporting HCMV miRNAs in extracellular vesicle-related fractions (Ding et al., 2017), our findings support the possibility that extracellular particles may contribute to the circulation of HCMV miRNAs. Nevertheless, the present data do not establish systemic EV-mediated transport, and future studies are needed to determine the relative contribution of EVs, ribonucleoprotein complexes, lipoproteins, virions, dense bodies, or other extracellular carriers. Moreover, our present study used a candidate-miRNA RT-qPCR approach rather than unbiased small RNA sequencing. Therefore, we cannot exclude the possibility that additional HCMV miRNAs or host miRNAs in plasma EV-enriched preparations may show stronger diagnostic or biological relevance. Future studies using small RNA sequencing followed by independent RT-qPCR validation are needed to comprehensively define the HCMV miRNA landscape in EV-enriched plasma particle fractions during HCMV DNAemia and DNA-low states.

Beyond their biomarker potential, several of the HCMV miRNAs detected in this study have reported biological roles relevant to HCMV pathogenesis. Hcmv-miR-US4-5p has been functionally linked to apoptosis regulation during HCMV infection. Previous studies identified PAK2 as a direct target of hcmv-miR-US4-5p using hybrid PCR and luciferase reporter assays. Overexpression of hcmv-miR-US4-5p reduced PAK2 protein levels in HEK293, HELF, and THP-1 cells, while naturally infected HELF cells showed a time-dependent decrease in PAK2 expression that coincided with increased hcmv-miR-US4-5p levels. PAK2-specific siRNA and hcmv-miR-US4-5p inhibitor experiments further demonstrated that hcmv-miR-US4-5p promotes apoptosis by repressing PAK2. These findings suggest that hcmv-miR-US4-5p may regulate host-cell apoptosis and modulate the balance between viral replication and host responses during natural HCMV infection (Shao et al., 2017). Hcmv-miR-UL36 may also be biologically relevant to HCMV replication and latency. As the only known intronic miRNA encoded by HCMV, hcmv-miR-UL36 overexpression increased HCMV DNA synthesis by more than threefold at 24 h post-infection. Mechanistically, UL138, a gene associated with HCMV latency, was identified as a target of hcmv-miR-UL36 by hybrid PCR and validated using luciferase reporter assay and western blotting. Hcmv-miR-UL36 reduced UL138 reporter activity by 74.6% and decreased UL138 protein expression by 46.2%, indicating that it may support HCMV replication by repressing the latency-associated UL138 gene (Huang et al., 2013). Hcmv-miR-US25-1-5p has been implicated in the regulation of HCMV replication. This miRNA is highly expressed during both lytic and latent infection and has been reported to inhibit viral replication. Functional target analysis identified multiple cellular transcripts as potential targets of hcmv-miR-US25-1-5p, including YWHAE, UBB, NPM1, and HSP90AA1, which were validated by luciferase reporter assay and western blotting. Because these host genes are involved in protein regulation, stress responses, and signaling pathways, hcmv-miR-US25-1-5p may influence HCMV replication by modulating host-cell pathways that directly or indirectly affect the viral life cycle (Jiang et al., 2015). Clinical evidence further suggests that these HCMV miRNAs vary according to infection stage and HIV coinfection status. In individuals with acute HIV infection coinfected with HCMV, hcmv-miR-US25-1-5p, hcmv-miR-US25-2-3p, hcmv-miR-US4-5p, hcmv-miR-US5-2-3p, and hcmv-miR-UL36 were more frequently detected during HCMV DNA-positive lytic infection. These findings suggest that these miRNAs may contribute to HCMV-HIV interactions by reflecting or influencing viral replication, immune activation, and inflammatory responses (Lazzari et al., 2025). Together, these studies support the biological relevance of the upregulated HCMV miRNAs identified in our EV analysis. Their detection in EV-enriched plasma particle fractions during HCMV DNAemia suggests that these miRNAs may be released into circulation in association with extracellular particles or other co-isolated carriers. Whether they are protected within bona fide EVs, delivered to recipient cells, or functionally contribute to viral replication, persistence, or immune modulation remains hypothesis-generating and requires further functional validation.

The kinetics of extracellular miRNA release, carrier association, and clearance in clinical samples are likely complex. *In vitro* studies show that HCMV miRNAs are detectable in EVs as early as 6 h post-infection and increase as the viral replication cycle progresses (Zhang J. et al., 2020). Our longitudinal analysis provides preliminary clinical observations regarding the dynamics of hcmv-miR-US25-1-3p in EV-enriched plasma particle fractions during antiviral therapy. We observed that changes in hcmv-miR-US25-1-3p levels in the EV-enriched plasma particle fraction appeared to lag behind changes in HCMV DNA load in several patients. The mechanism underlying this lag warrants further investigation but may be related to the kinetics of extracellular miRNA release, carrier association, and clearance from circulation. In some cases, we noted an inverse relationship, where miRNA levels rose as viral load declined, which could reflect complex host-pathogen interactions or differential clearance rates. Our longitudinal analysis suggests that hcmv-miR-US25-1-3p levels in the EV-enriched plasma particle fraction can change during antiviral therapy. However, the longitudinal component provides only preliminary descriptive observations. The seven treated patients had heterogeneous underlying diseases and showed inconsistent temporal relationships between hcmv-miR-US25-1-3p levels and HCMV DNA. Some patients showed divergent or inverse patterns, whereas one showed a more concordant decrease. These heterogeneous findings preclude conclusions regarding treatment monitoring. Larger prospective cohorts with standardized sampling intervals, independent validation, and better-defined clinical endpoints would be required to determine whether particle-fraction-associated HCMV miRNAs provide information complementary to HCMV DNA monitoring.

The ROC analysis yielded modest AUC values and low sensitivities. In addition, the same DNA-defined cohort was used for both candidate marker selection and ROC evaluation. Therefore, the diagnostic performance estimates are exploratory and may be optimistic. These miRNAs should not be considered standalone diagnostic biomarkers at this stage. Their potential value may lie in combination with HCMV DNA load, host immune or inflammatory markers, and clinical variables in future independently validated multi-marker models.

A major limitation of our study is the use of a precipitation-based particle-enrichment method. Although TEM and NTA confirmed recovery of vesicle-like nanoparticles, these methods do not establish EV identity, purity, or intravesicular miRNA localization. ExoQuick-based precipitation can co-isolate non-vesicular components, including protein aggregates, lipoproteins, ribonucleoprotein complexes, and potentially virus-related particles. We did not perform biochemical analysis for EV-positive markers, such as CD9, CD63, CD81, or TSG101, negative contamination markers, such as albumin or ApoA1, or RNase/protease protection assays to confirm whether the detected miRNAs were protected within membrane-enclosed vesicles. Therefore, our findings should be interpreted as HCMV miRNA signals detected in an EV-enriched plasma precipitate rather than definitive EV-encapsulated cargo. Future studies should use more stringent isolation approaches, such as size-exclusion chromatography, density-gradient purification, immuno-capture, or nano-flow cytometry, combined with EV-marker validation and nuclease/protease protection assays to define the precise carrier subtype. Moreover, other limitations of our study include the relatively small sample size, and the cross-sectional design of the main cohort. Furthermore, technical refinements for future studies include the use of exogenous spike-in controls to monitor RNA extraction efficiency. Despite these limitations, our findings show that selected HCMV miRNAs are increased in an ExoQuick-derived EV-enriched plasma particle fraction during HCMV DNAemia. Further studies are required to confirm whether these miRNAs are truly EV-encapsulated and to define their biological and clinical significance.

## Conclusion

In conclusion, this targeted descriptive study shows that selected HCMV-encoded miRNAs, particularly hcmv-miR-US4-5p and hcmv-miR-US25-2-5p, are increased in an ExoQuick-derived plasma particle precipitate from patients with HCMV DNAemia compared with DNA-low, IgG-positive controls. Because EV identity and miRNA encapsulation were not confirmed, these findings should be interpreted as particle-fraction-associated HCMV miRNA signals rather than definitive EV-encapsulated miRNAs. The exploratory ROC analyses showed modest diagnostic performance, indicating that these miRNAs are unlikely to function as standalone diagnostic tools. Future studies should validate these findings in independent cohorts and define the precise extracellular carriers using EV-marker analysis, more stringent isolation methods, and nuclease/protease protection assays.

## Data Availability

The original contributions presented in this study are included in this article/supplementary material. Further inquiries can be directed to the corresponding author.
